# The interaction of *Schistosoma mansoni* infection with diabetes mellitus and obesity in mice

**DOI:** 10.1038/s41598-023-36112-5

**Published:** 2023-06-09

**Authors:** Alaa S. Amer, Ahmad A. Othman, Lamees M. Dawood, Kholoud A. El-Nouby, Geoffrey N. Gobert, Dina M. Abou Rayia

**Affiliations:** 1 Medical Parasitology Department, Faculty of Medicine, Tanta University, Tanta, 31527 Egypt; 2 Biochemistry Department, Faculty of Medicine, Tanta University, Tanta, 31527 Egypt; 3 School of Biological Science, Institute for Global Food Security, Queen’s University Belfast, Belfast, BT9 5DL UK

**Keywords:** Parasitic infection, Parasitic infection

## Abstract

Human schistosomiasis is one of the most prevalent parasitic diseases worldwide. Various host factors can affect the host–parasite interactions. Therefore, the aim of the present work was to determine the parasitological, histopathological, biochemical, and immunological status of *Schistosoma mansoni*-infected hosts with metabolic disorders to identify the underlying possible mechanisms of these comorbidities. The study animals were divided into four groups. Group I represented the control groups, namely, the normal control group, the *S. mansoni*-infected control group, and the noninfected type 1 diabetes (T1DM), type 2 diabetes (T2DM), and obesity groups. The mice of the other three groups underwent induction of T1DM (Group II), T2DM (Group III) and obesity (Group IV) before being infected with *S. mansoni*. All mice were subjected to body weight measurement, blood glucose and insulin assessment, parasitological evaluation of adult worm count, tissue egg count and intestinal oogram. Histopathological and immunohistochemical study using anti-glial fibrillary acidic protein (GFAP) in hepatic stellate cells (HSCs) and image analysis of Masson’s trichrome-stained liver sections using ImageJ (Fiji) software were carried out. Additionally, immunological analysis of tumour necrosis factor (TNF) beta, interleukin-5 (IL-5), IL-10, Forkhead box P3 (FOXP3) and pentraxin 3 (PTX3) levels besides biochemical study of total lipid profile were evaluated. The present study revealed a significant increase in the adult worm count and tissue egg output in the obesity group compared to the infected control group. The oogram of counted eggs showed prevalence of immature eggs in T1DM group, while T2DM and obese groups showed prevalence of mature eggs. The fibrosis area percentage showed significant increase in T2DM and obese groups while it was decreased in T1DM group in comparison to infected control group. Our data also showed significant increase in the levels of TNF-β, IL-5, PTX3 in T1DM, T2DM and obesity groups in comparison to infected control group, whilst the levels of FOXP3 and IL-10 were increased in the infected groups in comparison to their noninfected controls. Moreover, infected T1DM, T2DM and obesity groups showed higher blood glucose and lipid profile in comparison to the infected control group. However, these parameters were improved in comparison to their noninfected controls. In sum, induction of T2DM and obesity increased tissue egg counts, mature egg percentage, and fibrosis density, while schistosome infection induced changes in the lipid profile and blood glucose levels in infected diabetic and obese groups and impacted favorably insulin levels in obese mice. By better understanding the complexities of host–parasite interactions, efforts to reduce the burden of these debilitating diseases can be improved.

## Introduction

Human schistosomiasis is one of the most prevalent parasitic diseases worldwide. It results in substantial levels of human morbidity and mortality in endemic areas, with an estimated 258 million people currently infected^1^. Globally, the disease contributes to 3.3 million disability-adjusted life years (DALYs)^2^. *Schistosoma mansoni* (*S. mansoni*) represents one of the major aetiological causes of schistosomiasis in Africa and South America^3^. Schistosomes are blood-dwelling flukes that are highly dependent on host metabolism. Several host-derived factors, such as interleukin-7 (IL-7), thyroid hormones and tumour necrosis factor (TNF), have been described to influence worm growth and fecundity. Therefore, it is important to consider the changes of normal host metabolism that could result in potential disruption of parasite oviposition and/or development^4^.

Type 1 diabetes mellitus (T1DM) or insulin-dependent diabetes mellitus is a metabolic disease characterized by the reduction or total absence of insulin production leading to high blood glucose concentrations. The disease is associated with some defects in cell-mediated immunity and individuals are reported to be more susceptible to microbial infections^4^. Adult schistosomes express insulin receptors (IRs) at the tegument basal membrane and in muscles. These receptors are involved in many biological processes including glucose uptake, parasite growth, and metabolism^5^. Therefore, the absence of host insulin may negatively impact parasite growth and reproduction^6^.

Researchers have reported that T1DM in mice with schistosomiasis may affect the cell-mediated immune response to parasite eggs. Smaller-sized granulomas around the eggs both in the liver and the intestine have been found, with fewer eggs excreted in faeces because of impaired egg maturation. However, the worm load and total number of eggs in the intestine tissue were equivalent to those of the control group^7^.

As T1DM is a Th1-mediated autoimmune disease, any skewing or correction of the immune response towards Th2, would result in diabetes prevention. *S. mansoni* soluble egg antigens have the potential to profoundly regulate the immune system of the infected host and prevent T1DM in nonobese diabetic (NOD) mice^8^. These responses are thought to occur due to prolonged production of immunoregulatory cytokines such as IL-4, IL-5, IL-10 and TGFβ. Moreover, it is thought that regulatory T cells (Tregs) may also affect glucose metabolism through their long-term secretion of IL-10 or transforming growth factor β (TGF-β), which inhibit the production of inflammatory cytokines or counteract the TNF-mediated inhibition of insulin signalling in adipocytes^9^. Hence, the comorbidities of T1DM and schistosomiasis may affect the consequences of both diseases.

Obesity results from an imbalance in food intake, basal metabolism, and energy expenditure. Sedentary behavior, like physical inactivity, also plays a role. In obese individuals, the amounts of nonesterified fatty acids, glycerol, hormones, cytokines and proinflammatory markers are all increased, potentially resulting in the development of insulin resistance, and the function of β-islet cells of the pancreas is impaired^10^. Thus, obesity is considered a high-risk factor for the development of type 2 diabetes mellitus (T2DM).

Adult schistosomes are fully dependent on host fatty acids as they do not have the capacity for de novo synthesis during their intra-mammalian life cycle stages. This aspect of schistosome parasitism has been postulated to be potentially beneficial to the host by reducing lipid accumulation in blood vessels, improving obesity, and decreasing its complications, including T2DM^11^. The pathophysiology of T2DM remains unclear and contentious. The onset of hyperglycaemia in mammals is characterized by three primary defects: resistance to insulin in peripheral tissues, increased hepatic gluconeogenesis and impaired insulin release^12^. Cholesterol is another host factor actively involved in the modulation of parasite cell signalling, and reproduction and may increase the rate of egg laying of worms in mice fed high-fat chow^13^. While schistosomiasis may potentially protect the host from some aspects associated with obesity complications, the impact of increased liver fibrosis is an obvious concern^14^.

Therefore, the aim of the present work was to study the interaction between *S. mansoni* infection and T1DM, T2DM, and obesity. We report on parasitological, histopathological, biochemical, and immunological parameters in a mouse model of hepatic schistosomiasis to identify the underlying possible mechanisms of the pathology resulting from these comorbidities.

## Materials and methods

### Animals

Two hundred parasite-free male black mice (C57BL/6), aged 4‒5 weeks, and weighing 20‒25 gm each, were obtained from Theodor Bilharz Research Institute, Giza, Egypt. They were maintained in cages (20 mice in each cage) with controlled temperature (22 ± 2 °C), humidity (55% ± 10%), and with continuous air renovation. The animals were housed in a 12 h light/12 h dark cycle (6AM‒6 PM). Animals were sacrificed using an approved method of euthanasia^4^.

### Parasite

*Biomphalaria alexandrina* snails infected with *S. mansoni* were obtained from the Biological Unit, Theodore-Bilharz Research Institute, Giza, Egypt. Cercarial shedding was performed, and mice were infected by subcutaneous injection with 40 ± 10 cercariae, as described by Peters and Warren^15^.

### Animal models

#### Induction of T1DM

T1DM was induced by intraperitoneal injection of a single dose (180 mg/kg) of streptozotocin (STZ) (Biokit, Victoria, Alexandria, Egypt) dissolved in freshly prepared 40 mM citrate buffer (0.410 gm citric acid and 0.558 gm k citrate in 100 ml distilled water) (pH 4.5) as described previously^4^. Mice with blood glucose exceeding 180 mg/dl were considered diabetic and thus were selected.

#### Induction of T2DM

Mice were fed high-fat chow containing 35.5% lipid, 20% protein and 36.6% carbohydrate for a period of 3 weeks. Then, they were injected intraperitoneally with a single dose of 100 mg/kg body weight STZ and kept on the same diet for the next 4 weeks as described previously^16^. Mice with a blood glucose level exceeding 180 mg/dl were considered diabetic and thus were selected.

#### Induction of obesity

Mice were fed a high-fat chow containing 35.5% lipid, 20% protein and 36.6% carbohydrate for a period of 5 months until increasing their weight by 50% from their starting level. The ingredients were purchased from commercial sources and were prepared weekly and stored at + 4 °C as described previously^16,17^.

### Experimental design

Group I (control) included the following subgroups: healthy (normal control) where mice were kept uninfected and were fed a commercial standard chow containing 12% fat, 28% protein and 60% carbohydrate as described previously^14^, and water and food were freely available; infected control where mice were infected with *S. mansoni* cercariae as described previously^15^; and noninfected T1DM, T2DM and obesity control subgroups. All mice were sacrificed for comparative study at the end of the experiment. Group II (infected T1DM): T1DM was induced as described previously. Seven days later, mice were infected with *S. mansoni* cercariae. Group III (infected T2DM): T2DM was induced as described previously. Then mice were infected with *S. mansoni* cercariae. Group IV (infected obesity): Obesity was induced as described previously. Then, the mice were infected with *S. mansoni* cercariae.

All animal groups were subjected to total body weight weekly measurement, and food intake was monitored daily, determined by the difference between the food supplied and the amount of food left in the cage. The food was renewed daily, and the remaining chow was discarded. All mice were euthanized in week 8 after cercarial challenge with a 0.05 mg/gm sodium pentobarbital intraperitoneal injection^18^.

### Parasitological parameters

#### Total worm burden

This was assessed by counting all recovered worms from mesenteric blood vessels, the portal vein, and the liver. A small slit was made in the hepatic portal vein, and then a needle was inserted into the descending aorta. Perfusion fluid was pumped through the needle, and the perfusate was collected in a container. The worms were collected from the perfusate and transferred by a Pasteur pipette into Petri dishes, washed with normal saline, and count / mouse was estimated according to Duvall and DeWitt^19^.

#### Tissue egg count

*Schistosoma mansoni* eggs were counted in the liver, and small and large intestinal tissues (from the stomach-duodenum junction to the middle part of the rectum and into the pelvic cavity). The livers and intestines of the mice were taken after perfusion. Approximately 0.5 gm of the liver and colon of each mouse were weighed and used to estimate the number of *S. mansoni* ova by the KOH digestion method described by Cheever^20^. The large intestines of the mice were digested in 4% KOH and centrifuged (2000 rpm for 5 min). Five aliquots of 50 µl of the digested tissue were placed on a slide and covered with a coverslip before counting eggs by light microscopy^21^.

#### Oogram

A quantitative oogram was performed for different developmental egg stages. Briefly, a 1 cm long sample from the last part of the small intestine was taken, opened lengthwise and crushed between two glass slides to obtain a thin preparation. The percentage of eggs at various developmental stages (immature, mature and dead) was determined by light microscopy according to Cançado et al.^22^.

### Histopathological examination

Animal livers and intestines were taken after perfusion and divided into roughly equal portions weighing 300‒400 mg. These portions were randomly assigned to the test groups and weighed to approximately 2 mg. All unfixed tissues were stored at − 20 °C prior to digestion as described in parasitological parameters. Fixed tissues from all the studied groups were left for 4‒120 days in 10% formalin and paraffin blocks were made according to Bancroft and Stevens^23^. Serial Sects. (5 µm) were cut and stained with haematoxylin and eosin to detect the pathological changes in the hepatic parenchyma, and with Masson’s trichrome stain to determine the amount of fibrosis present. Granuloma size was determined by using optical micrometer; data are presented as mean cross-sectional diameter ± standard error (SE). Bright field microscopy was used to determine the density of total granuloma formation and the number of each stage in an area of 1 mm^2^. According to Lenzi et al.^24^, granulomas were divided into exudative (E), exudative/exudative‒productive (E/EP), exudative‒productive (EP), and productive (P) stages. The liver fibrosis percentage was measured in the liver by an image analyser according to Abdalla et al.^25^. ImageJ (Fiji) was used for analysis. Using Fiji's deconvolution plugin, the RGB images were separated into three components: red, blue, and green. ImageJ (Fiji) was used to compute the area and “integrated density” (IntDen), which is the product of the area and mean intensity^26^.

### Immunohistochemical analysis

Immunohistochemical staining of hepatic stellate cells (HSCs) was performed using glial fibrillary acidic protein (GFAP) according to Gibelli et al.^27^. Immunohistochemical staining was performed on 3–5 µm sections from 10 randomly selected paraffin blocks from each of the infected groups, using the Ultra Vision Detection System (Anti-Polyvalent, HRP/DAB ‘‘Ready-to-Use’’, Cat. #TP-015-HD, Lab Vision, USA) and antibody against GFAP (Ab-4 rabbit polyclonal antibody, Cat. #RB-087-R7 ‘‘Ready-to-Use’’, Lab Vision, USA). The procedure was conducted according to the manufacturer’s protocol. Staining was viewed with a biotinylated goat anti-polyvalent antibody (secondary antibody) and streptavidin peroxidase. In each slide, the percentage of positive HSCs to the total number of HSCs was calculated. Each group's average percentage of activated HSCs (positively stained) was then calculated. Semiquantitative grading of HSCs immunostained with GFAP was determined using scores from 0 to III, with 0 indicating the absence of positive staining or less than 3% staining; I indicating 3–33% positive staining; II indicating 34–66% positive staining; and III indicating more than 66% positive staining^28^.

### Immunological parameters

Determination of the following immunological indices was performed using the plasma of all sacrificed mice by ELISA^29^. TNF beta is a marker for Th1 responses; IL-5 and IL-10 are markers for Th2 responses; Forkhead box P3 (FOXP3) is a marker for T regulatory cells; and pentraxin 3 (PTX3) in plasma is a marker for inflammation. The kits were purchased from Biokit, Victoria, Alexandria, Egypt. They are based on the use of a double-antibody sandwich ELISA to determine the level of mouse-specific markers in the samples. The chroma of colour and the concentration of the mouse substance of the sample were positively correlated by taking the blank well as zero and measuring the optical density (OD) under a 450 nm wavelength which should be carried out within 15 min after adding the stop solution. According to the standard concentrations and the corresponding OD values, the standard curve linear regression equation was calculated, and then the OD values of the samples were applied to the regression equation to calculate the corresponding sample concentrations.

### Biochemical analyses

Mice fast overnight for 8‒12 h. Blood samples were obtained by puncture of the retro-orbital sinus. The fasting blood glucose levels were measured on a Thermo Fisher Sr. Konelab clinical chemistry analyser using standard automated enzymatic methods.

Plasma samples were separated by centrifugation at 120 × g for 15 min and stored at − 80 °C for lipid profile analysis. Plasma low-density lipoprotein (LDL), high-density lipoproteins (HDL), and triglycerides (TG) were all measured to obtain a complete lipid profile. The colorimetric enzymatic approach was used to extract all the lipids. Total cholesterol (TC) was determined by the cholesterol esterase/cholesterol oxidase/peroxidase method as described by the National Cholesterol Education Program (NCEP) expert panel^30^. The glycerol phosphate oxidase/peroxidase technique was used to determine TG as described previously^31^ using a triglyceride kit purchased from Sigma Aldrich. The concentration of plasma HDL was estimated by a colorimetric kit following the method of Finley et al.^32^ using an HDL kit purchased from SPINREACT, S.A. Ctra, Santa Coloma, Spain. The LDL concentration was calculated from the total cholesterol concentration, HDL concentration, and TG concentration according to Friedewald et al.^33^.

Insulin levels were measured in the plasma of all scarified mice by ELISA using kits purchased from Biokit, Victoria, Alexandria, Egypt, as described in immunological parameters.

### Statistical analysis

The statistical analysis used included ordinary one-way ANOVA for the ova count, immunological marker levels as well as insulin and glucose levels. Two-way ANOVA was used for assessment of body mass, total worm burden, oograms, lipid profiles, and granulomas and to compare Masson trichrome image analysis and GFAP expression between groups. Results are presented as the mean ± standard deviation for each group using GraphPad Prism 9. *P* values less than 0.05 were regarded as significant.

### Ethics approval

All methods used in this study are in accordance with ARRIVE guidelines. The Animal care and experimental protocol were reviewed and approved in accordance with guidelines of the local ethics committee for care and use of animal in a humane manner under the approval protocol number 33388/08/19 by Tanta University, Faculty of Medicine, Research Ethics Committee, Federal Wide Assurance (FWA), FWA00022834, IRB0010038.

## Results

### Body mass assessment

**Figure 1:**
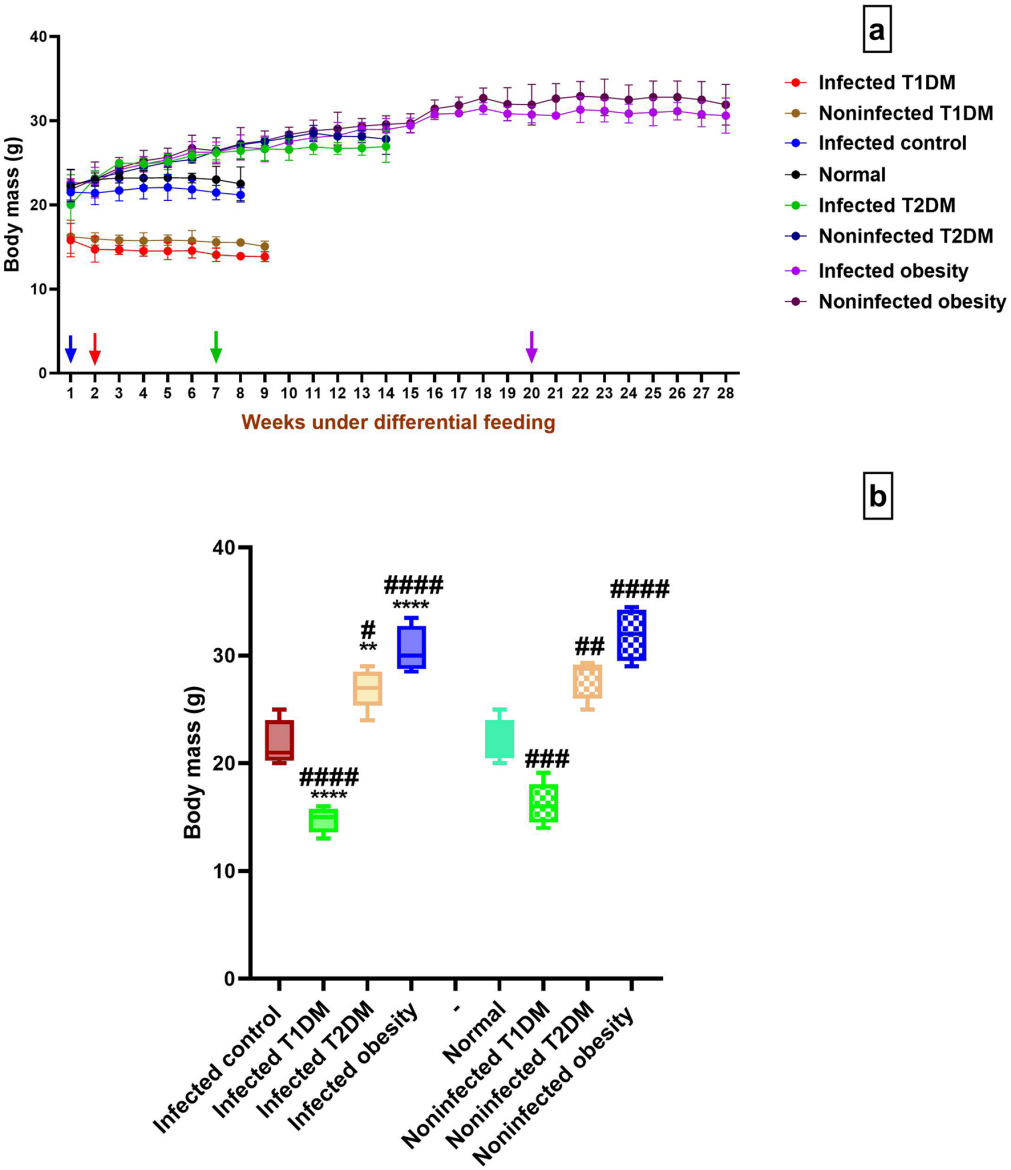
Body mass variation. (**a**) during the experiment with colored arrows pointing at the week of infection in each of the infected groups. (**b**) at the end of the experiment. **P < 0.01, ****P ˂ 0.0001 compared to the infected control group. #*P* < 0.05, ###*P* ˂ 0.001, ####*P* ˂ 0.0001 compared to the normal group. (n = 5 per group). T1DM: type 1 diabetes mellitus; T2DM: type 2 diabetes mellitus.

The infected T1DM group showed significantly lower body mass than the infected control group. The infected T2DM and obesity groups showed significantly higher body mass than the infected control group. There were no significant differences between the infected groups and the noninfected groups in the T1DM, T2DM, and obesity models (*P* > 0.05). There were significant differences between the infected and noninfected diabetic and obesity groups and the normal group (Fig. 1).

### Parasitological parameters

Results of the worm recovery from infected groups showed that DM had no effect on the worm recovery compared to the control group. Obesity significantly increased the worm burden (*P* = 0.0005; Table 1).

**Table 1:** Adult worm count recovered from the infected mice.

Groups	Male worm liver		Female worm liver		Total worm liver		Male worm intestine		Female worm intestine		Total worm intestine		Mean total worms	
	Mean	SE	Mean	SE	Mean	SE	Mean	SE	Mean	SE	Mean	SE	Mean	SE
Infected control	6.3	0.4	3.7	0.5	9.1	0.8	9.5	0.4	5	0.26	13.5	0.1	21.6	0.8
Infected T1DM	6.2	0.43	3.11	0.32	8.3	0.7	8.5	0.9	4.3	0.3	12.7	1.3	20.2	1.6
Infected T2DM	6.22	0.32	3.21	0.39	8.7	0.71	9.3	0.6	4.8	0.23	13	1.1	21.1	0.7
Infected obesity	7.1	0.37	4.2	0.5	11.1	0.76	11.7	0.42	6.6	0.34	15	0.3	23.6***	0.4

Results are given as mean ± standard error, ***P < 0.001 compared with the infected control group. (n = 20 per group). T1DM, type 1 diabetes mellitus; T2DM, type 2 diabetes mellitus.

**Figure 2:**
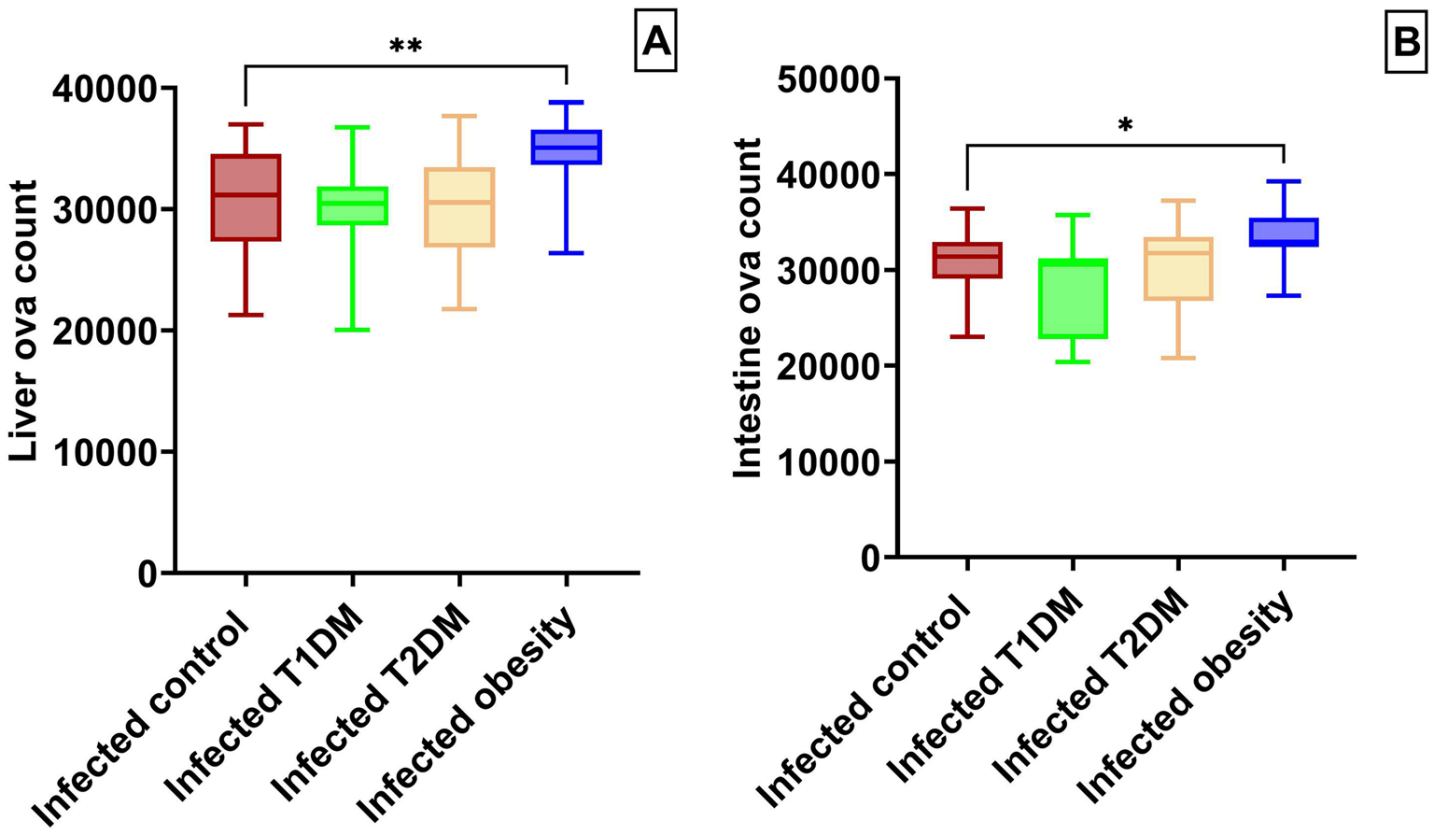
Ova count in infected mice groups. (**a**) liver ova count. (**b**) intestine ova count. *P < 0.05, **P < 0.01. (n = 20 per group). T1DM: type 1 diabetes mellitus; T2DM: type 2 diabetes mellitus.

Liver egg counts were used as an indicator of pathology for the different animal models of schistosomiasis. In comparison with the infected control group, there was a significant increase in the hepatic ova count in the obesity group (F _(3, 76)_ = 6.910, *P* = 0.0038; Fig. 2a). As an indicator of potential disease transmission, intestinal ova counts were also determined. There was a significant increase in the intestinal ova count in the obesity group compared with the infected control group (F _(3, 92)_ = 9.484, *P* = 0.0306; Fig. 2b).

**Figure 3:**
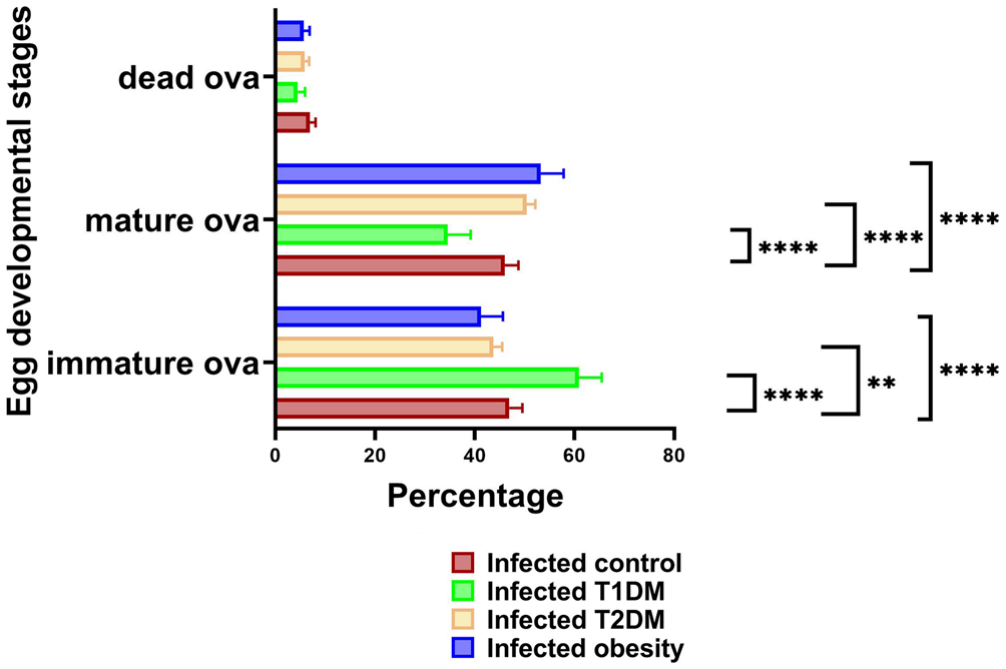
Mean percentage of oogram in infected groups. **P < 0.01, ****P ˂ 0.0001. (n = 20 per group). T1DM: type 1 diabetes mellitus; T2DM: type 2 diabetes mellitus.

The oogram showed a significant increase in immature ova percentage in the T1DM group and a significant reduction in the T2DM and obesity groups compared with the infected control group. For the mature ova percentage, there was a significant decrease in the T1DM group. Additionally, it was increased in the T2DM and obesity groups. For the dead ova percentage, there were no significant differences between groups (Fig. 3).

### Histopathological and histochemical findings

Histological assessment demonstrated the presence of granulomatous responses within the portal tract. The granulomas were around the *Schistosoma* ova and were made up of a cellular assemblage of epithelioid cells, eosinophils, lymphocytes, histiocytes, and plasma cells. Peripheral fibroblasts with varying levels of collagen deposition were observed in both fibro-cellular and fibrous types. However, fibroblasts prevailed in both exudative-productive (EP) and productive (P) granulomas, while cellular components were present to a lesser degree.

**Figure 4:**
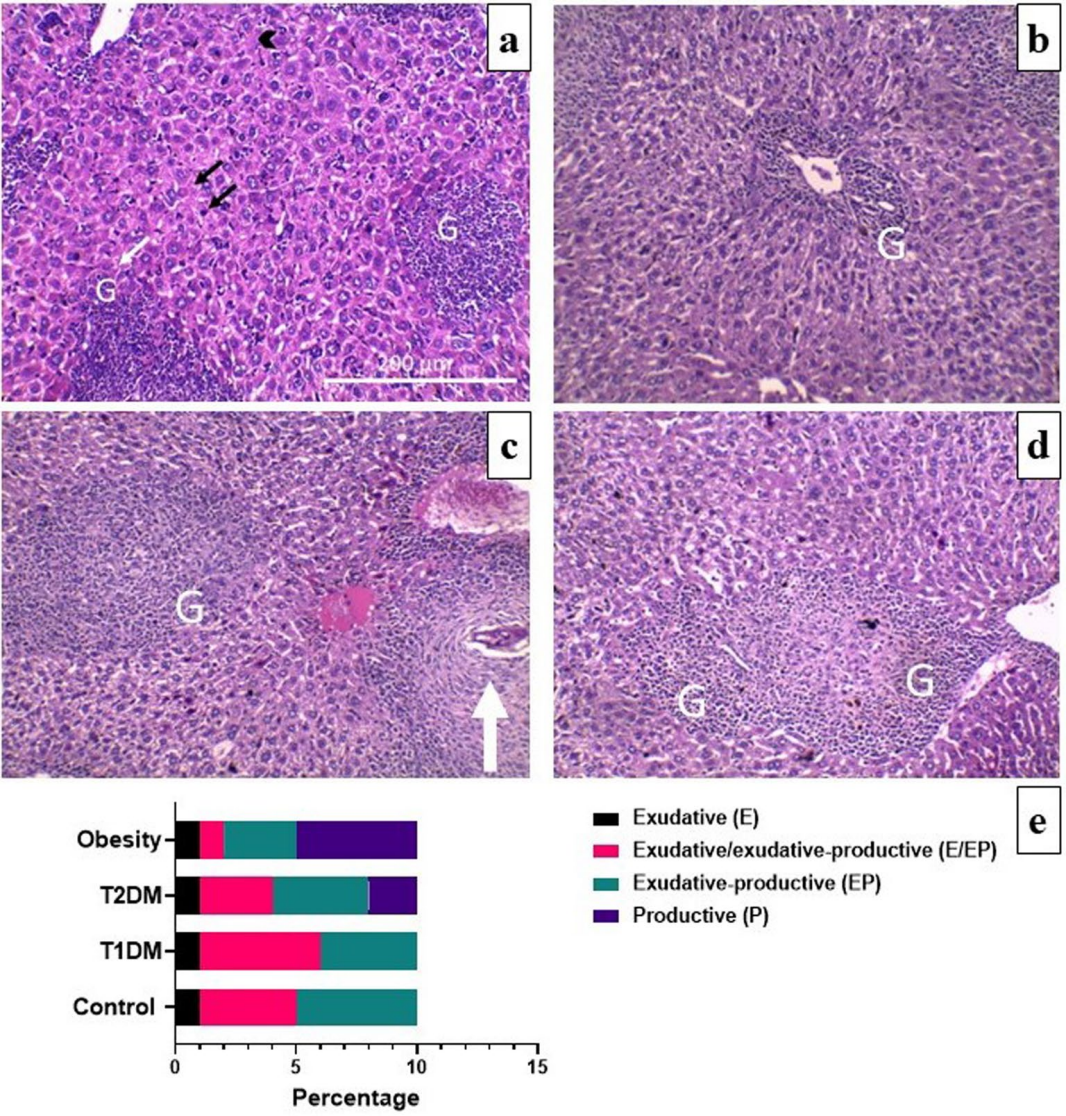
Histopathological changes of liver sections of *Schistosoma mansoni* infected mice (H&E). (**a**) in control groups showing exudative productive multiple granulomas (G) surrounded by concentric ring of epithelioid cells, eosinophils, and lymphocytes. Note the disorganized hepatic strands, dilated sinusoids, hyper eosinophilic hepatocytes (small black arrow), hypertrophied Kupffer cell (black arrowhead) and necrotic hepatocytes (small white arrow) adjacent to the granuloma. (**b**) in type 1 diabetes mellitus (T1DM) groups showing slightly reduced granuloma. (**c**) in type 2 diabetes mellitus (T2DM) groups showing exudative productive multiple granulomas with trapped central egg (large white arrow) with some degrees of macrosteatosis. (**d**) in obesity groups showing productive multiple granulomas with some degrees of macrosteatosis. (**e**) Percentage of different stages of hepatic granuloma formation / 1 mm^2^ of infected control, T1DM, T2DM, and obesity groups. There were no significant differences between control and infected groups, *P* > 0.05. (n = 10 per group).

The percentages of the various types of hepatic granulomas in the studied groups were determined. There were no significant differences between infected control and infected T1DM, T2DM and obesity groups (*P* > 0.05; Fig. 4). Exudative (E), exudative/exudative-productive (E/EP), EP, and P granulomas were found in the livers of infected obese mice, while the late stage was not found in the livers of infected control mice (Fig. 4a and d). Infected T1DM group showed slightly reduced size of granulomas (Fig. 4b), however, there were no significant differences in hepatic granuloma diameter (µm) between groups, *P* > 0.05 (Table 2). There was some degree of macrosteatosis in the T2DM and obesity groups (Fig. 4c and d). Macrovesicular steatosis caused by large lipid droplets (LDs) in the cytoplasm of hepatocytes is an expected feature in T2DM^34^. EP granulomas were the most common type in the infected control and T2DM groups (Fig. 4e). E/EP granulomas predominated in the infected T1DM group, while P granulomas were prevalent in the infected obesity group (Fig. 4e). There were significant differences in granuloma density/1 mm^2^ of the infected obese group as compared to infected control group, *P* < 0.01 (Table 2).

**Figure 5:**
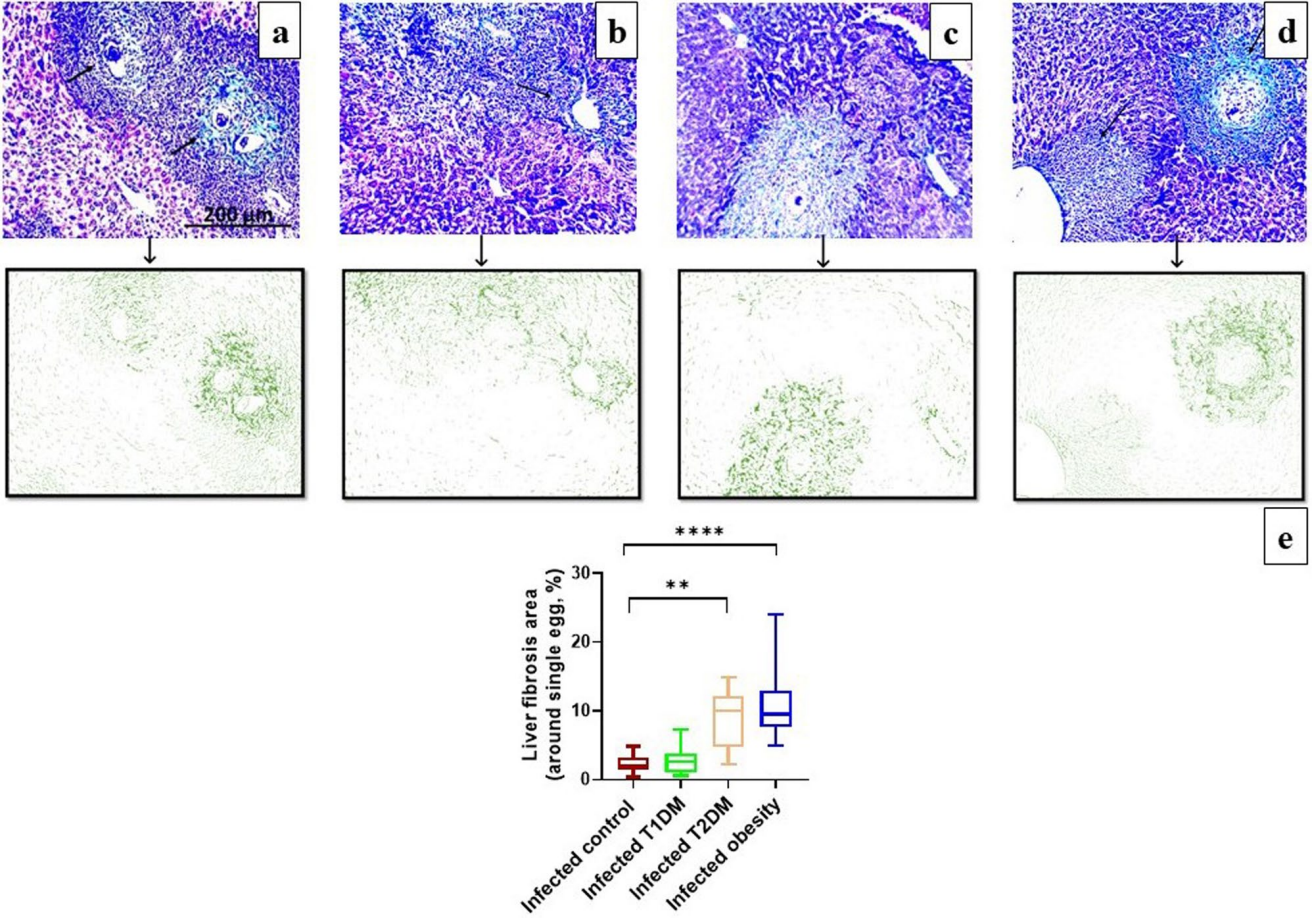
Photomicrographs of liver sections of *Schistosoma mansoni* infected mice (Masson’s trichrome). (**a**) in control groups showing high fibrotic changes within the granulomas (black arrow). (**b**) in type 1 diabetes mellitus (T1DM) group showing less fibrotic changes within the granuloma. (**c**) in type 2 diabetes mellitus (T2DM) groups. (**d**) in obesity group showing extensive fibrotic changes within the granulomas. By using Fiji's deconvolution plugin. The RGB images were separated into three components: red, blue, and green. In the green image, the collagen fibers from the area of interest were quantified. (**e**) The percentage of fibrotic area in the liver of infected groups. There were significant differences when comparing T2DM and obesity groups with the control group, **P < 0.01, ****P ˂ 0.0001. (n = 10 per group).

Masson's trichrome staining identified fibrotic changes within the granulomas. The collagen fiber densities from the area of interest were quantified in the green channel of the image. The fibrotic area percentage was calculated by dividing the collagen deposition area by the total area. There were significant increases when comparing the fibrosis area percentage in the T2DM and obesity groups with that in the infected control group (Fig. 5 and Additional file 1: Table S1).

**Figure 6:**
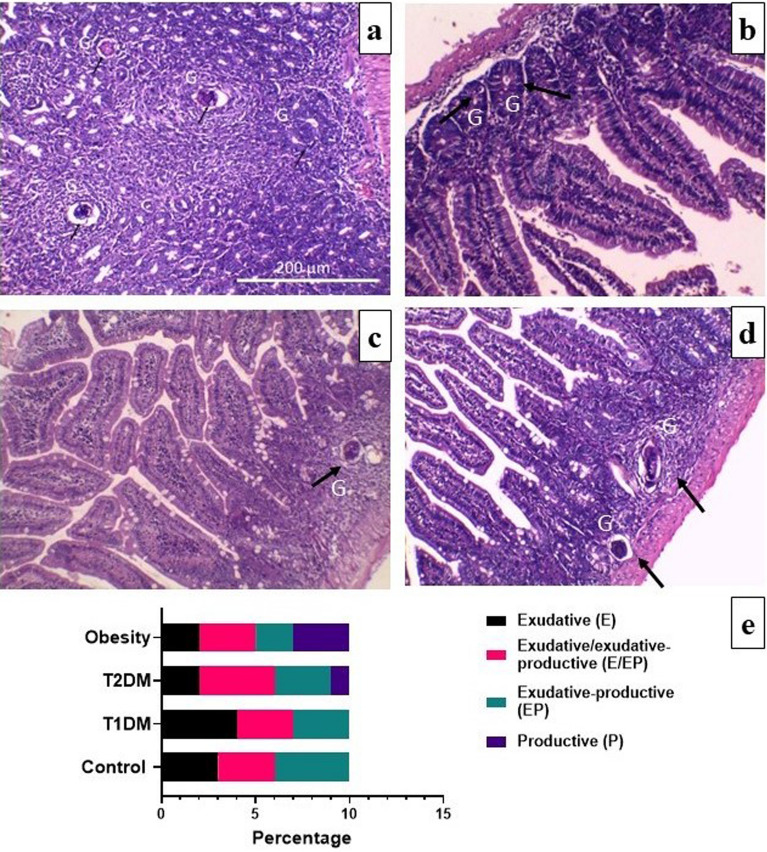
Histopathological changes of intestinal sections of *Schistosoma mansoni* infected mice (H&E). (**a**) in control group showing multiple granulomas (G) with trapped central egg (black arrow) surrounded by concentric ring of epithelioid cells, eosinophils, and lymphocytes. (**b**) in type 1 diabetes mellitus (T1DM) group showing remarkably reduced granulomas. (**c**) in type 2 diabetes mellitus (T2DM) group showing some degrees of macrosteatosis. (**d**) in obesity group showing multiple granulomas with some degrees of macrosteatosis. (**e**) Percentage of different stages of intestinal granuloma formation / 1 mm^2^ in the infected control, T1DM, T2DM, and obesity groups. There were no significant differences between control and infected groups, *P* > 0.05. (n = 10 per group).

Intestinal granulomas showed a marked reduction in size in the T1DM group (Fig. 6b). There was some degree of macrosteatosis in the T2DM and obesity groups (Fig. 6c and d). The percentages of the various types of intestinal granulomas in the studied groups were demonstrated. There were no significant differences between the infected control and infected T1DM, T2DM and obesity groups (*P* > 0.05; Fig. 6). There was a significant decrease in the intestinal granuloma diameter (µm) in the infected T1DM group compared to the infected control group, *P* < 0.01. There were significant differences in the granuloma density/1 mm^2^ of the infected obese group compared to the infected control group, *P* < 0.05 (Table 2).

**Table 2:** Granuloma diameter (µm) and density/1 mm^2^ of the infected mice.

	Infected control	Infected T1DM	Infected T2DM	Infected obesity
Liver granulomas				
Granuloma dimeter (µm)	240 ± 32	200 ± 26	250 ± 30	300 ± 21
Granuloma density/1 mm^2^	1 ± 0.2	0.7 ± 0.1	3.5 ± 0.21	10.2 ± 0.4**
Intestine granulomas				
Granuloma dimeter (µm)	230 ± 25	154 ± 20**	245 ± 54	289 ± 60
Granuloma density/1 mm^2^	0.9 ± 0.3	0.6 ± 0.2	2.3 ± 0.4	6.5 ± 0.1*

Results are given as mean ± standard error, *P < 0.05, **P < 0.01. (n = 10 per group). T1DM, type 1 diabetes mellitus; T2DM, type 2 diabetes mellitus.

### Immunohistochemical evaluation of GFAP expression

**Figure 7:**
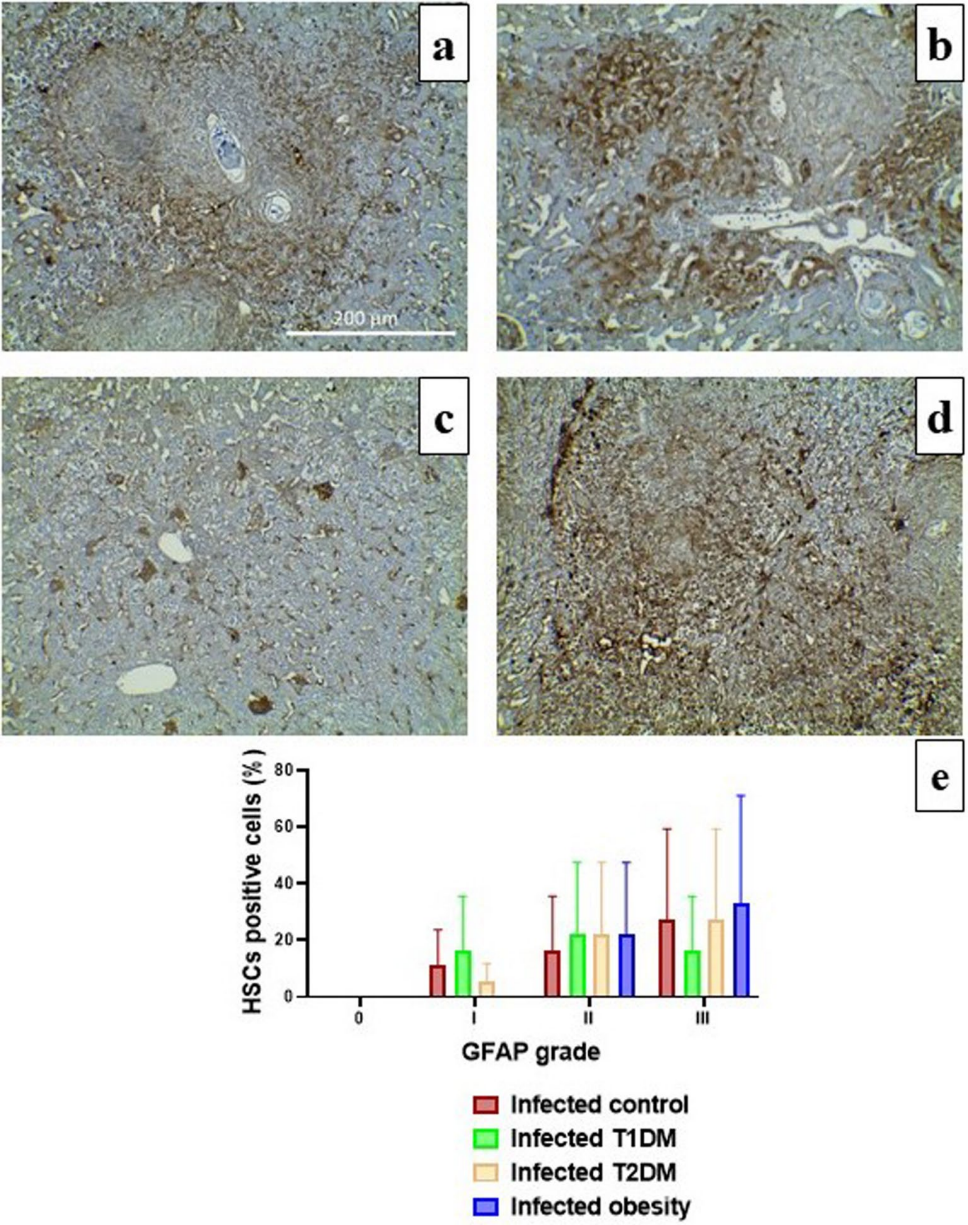
Immunohistochemical staining of glial fibrillary acidic protein (GFAP) in the livers of infected mice. (**a**) in control groups showing intensely immunostained hepatic stellate cells (HSCs). (**b**) in type 1 diabetes mellitus (T1DM) group showing moderate expression of GFAP. (**c**) in type 2 diabetes mellitus (T2DM) group showing moderate expression of GFAP. (**d**) in obesity group showing intense expression of GFAP and (**e**) The percentage HSCs positive for GFAP expression in the liver of infected groups. There were no significant differences between control and infected groups, *P* > 0.05. (n = 10 per group).

It was found that immunostained HSCs were abundant in the sinus walls and perivenular areas of the livers of obese mice, with 60% of the GFAP-immunostained HSCs being grade III. In contrast, grade II was predominant in the T1DM group, with 40% GFAP-immunostained HSCs. The infected control and T2DM groups showed 50% of the GFAP-immunostained HSCs being grade III. There were no statistically significant differences between the T1DM, T2DM and obesity groups and the infected control group (*P* > 0.05; Fig. 7 and Additional file 2: Table S2).

### Immunological parameters

**Figure 8:**
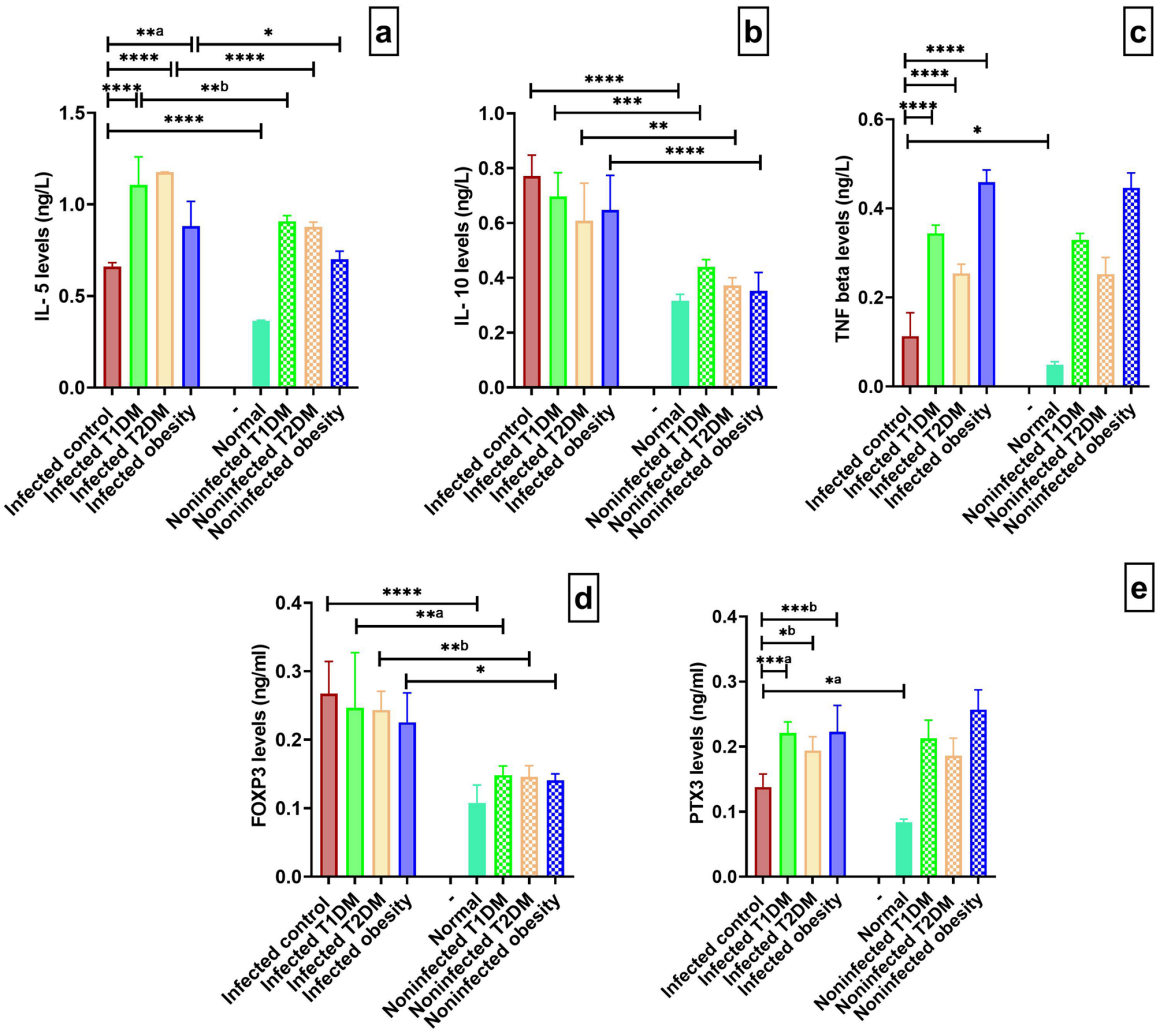
Immunological markers measured in infected and noninfected groups. (**a**) interleukin-5 (IL-5) levels (ng/L). (**b**) interleukin-10 (IL-10) levels (ng/L). (**c**) tumour necrosis factor (TNF) beta levels (ng/L). (**d**) forkhead box P3 (FOXP3) levels (ng/ml). (**e**) pentraxin 3 (PTX3) levels (ng/ml). *P < 0.05, **P < 0.01, ***P ˂ 0.001, ****P < 0.0001. (n = 5 per group). T1DM: type 1 diabetes mellitus; T2DM: type 2 diabetes mellitus.

Concerning IL-5 levels in the control groups, there was a significant increase in the infected control group compared to the normal (negative control) group. When comparing the levels in the infected T1DM, T2DM and obesity groups with the infected control group, there were significant increases in the T1DM and T2DM groups and in obesity group. In the T1DM, T2DM and obesity models, there were significant increases in the infected T1DM, T2DM and obesity groups compared to the noninfected groups (Fig. 8a).

IL-10 levels were significantly elevated in the infected control group compared with the normal group. There were no significant differences when comparing the infected groups with the infected control group (*P* > 0.05). In the T1DM, T2DM and obesity models, there were significant increases in the infected T1DM, T2DM and obesity groups compared to the noninfected groups (Fig. 8b).

Tumour necrosis factor beta levels were significantly increased in the infected control group in relation to the normal group. There were significant increases in the infected T1DM, T2DM and obesity groups compared with the infected control group (Fig. 8c). In the T1DM, T2DM and obesity models, there were no significant differences when comparing the infected T1DM, T2DM and obesity groups to the noninfected T1DM, T2DM and obesity groups (*P* > 0.05).

Regarding FOXP3 levels, compared to the normal group, the infected control group had a significantly increased level. There were no significant differences between the infected groups and the infected control group (*P* > 0.05). In the T1DM, T2DM and obesity models, there were significant increases in the infected T1DM, T2DM and obese groups compared to the noninfected groups (Fig. 8d).

Regarding PTX 3 levels, there was a significant increase when comparing the infected control group with the normal control group. There were significant increases in the infected T1DM, T2DM and obesity groups compared with the infected control (Fig. 8e). In the T1DM, T2DM and obesity models, there were no significant differences when comparing the infected T1DM, T2DM and obesity groups to the noninfected T1DM, T2DM and obesity groups (*P* > 0.05).

### Biochemical findings

**Figure 9:**
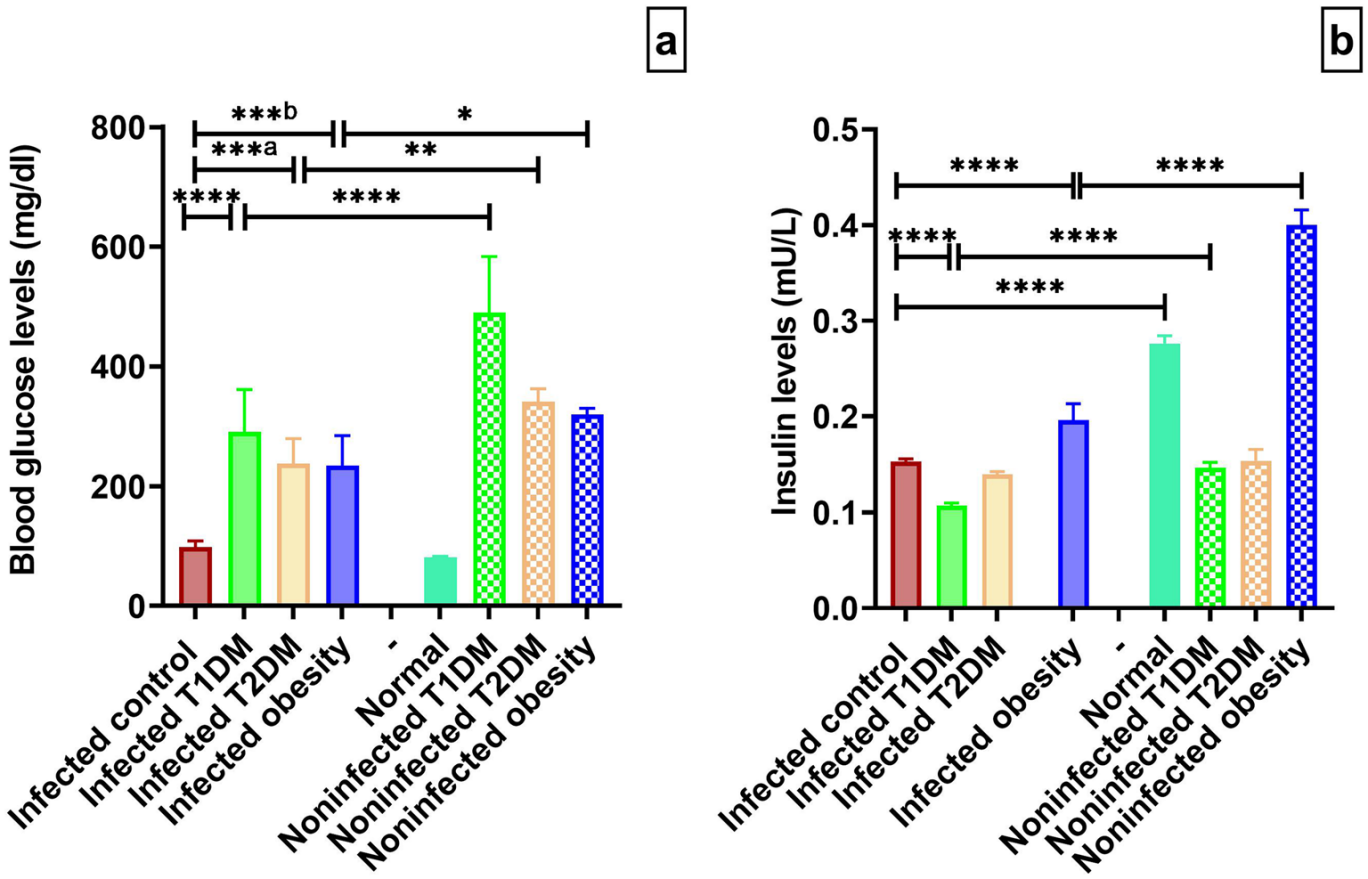
Metabolic parameters measured in infected and noninfected groups. (**a**) glucose levels (mg/L). (**b**) insulin levels (mU/L). *P < 0.05, **P < 0.01, ***P ˂ 0.001, ****P < 0.0001. (n = 5 per group). T1DM: type 1 diabetes mellitus; T2DM: type 2 diabetes mellitus.

Regarding blood glucose levels, there were significant increases in the T1DM, T2DM and obesity groups compared with the infected control group. There was no significant difference between the infected control group and the normal group (*P* > 0.05).There were significant decreases in glucose levels in the infected T1DM, T2DM and obesity groups compared to the noninfected T1DM, T2DM and obesity group (Fig. 9a).

In terms of the lipid profile in the infected groups compared with the infected control group, TG, HDL, LDL, and TC levels increased significantly in the T1DM, T2DM and obesity groups. There were no significant differences between the infected control group and the normal control group (*P* > 0.05). In the infected T1DM and obesity groups compared to the noninfected T1DM and obesity groups, there were significant decreases in LDL, TG and TC. There was a significant decrease in HDL and TC in the infected obesity group compared to the noninfected obesity group. There was a significant decrease in TG and TC in the infected T2DM group compared to the noninfected T2DM group (Table 3).

**Table 3:** Lipid profiles in infected and noninfected groups.

Lipid profile (mg/dl)	LDL			HDL			TG			TC		
Groups	Mean	SD	N	Mean	SD	N	Mean	SD	N	Mean	SD	N
Infected control	12.2	1.924	5	13.8	2.775	5	17.2	4.147	5	29.4	0.894	5
Infected T1DM	46**** ###^a^	4.359	5	47****	6.892	5	50.8**** ####	18.377	5	103.2**** ####	11.498	5
Infected T2DM	51.2****	6.38	5	42.8****	5.404	5	45.2**** ####	6.221	5	102.8**** ##	7.662	5
Infected obesity	33.4**** ###^b^	2.702	5	34.6**** #	3.362	5	34.4*** ###^c^	10.139	5	75**** ####	4.743	5
Normal	11	1.581	5	11.8	2.387	5	13.2	1.924	5	25.4	3.647	5
Noninfected T1DM	62.2	1.924	5	47.2	1.483	5	73.2	8.871	5	124.2	3.421	5
Noninfected T2DM	57.4	2.074	5	44.8	2.387	5	71.2	5.975	5	116.6	3.782	5
Noninfected obesity	50.4	3.647	5	46	2.236	5	50.2	1.924	5	106.6	2.881	5

***P ˂ 0.001, ****P < 0.0001 compared with the infected control group. ^#^ *P* < 0.05, ##*P* < 0.01, ###*P* < 0.001, ####*P* < 0.0001 comparing the infected T1DM, T2DM, and obesity groups to the noninfected diabetic and obesity groups. T1DM, type 1 diabetes mellitus; T2DM, type 2 diabetes mellitus; LDL, low-density lipoprotein; HDL, high-density lipoproteins; TG, triglycerides; TC: total cholesterol.

Insulin levels were significantly decreased in the infected control group compared to the normal group. There was a significant decrease in the T1DM group and a significant increase in the obesity group compared with the infected control group. There were no significant differences when comparing the infected T2DM group with the infected control group or the noninfected T2DM group (*P* > 0.05). The infected T1DM group had a significant decrease compared to the noninfected T1DM group. The levels in the infected obesity group were significantly lower than those in the noninfected obesity group (F _(7,32)_ = 475.0, ****P ˂ 0.0001; Fig. 9b).

## Discussion

Various host factors can affect host–parasite interactions. These factors can include changes in metabolism, immunological responses, and genetic background^35^. These changes in turn may disrupt important parasite functions such as oviposition, worm development and the resulting pathology associated with the infection^4^. In the current study, we determined the parasitological, histopathological, biochemical, and immunological status of *S. mansoni*-infected hosts with metabolic disorders. Through animal models of DM and obesity, we identified the underlying mechanisms of schistosome‒host interactions leading to the formation of pathology.

In this study, body mass showed significant differences between the study groups and controls with significant increase in the T2DM and obesity groups due to improved metabolic performance. These findings were in accordance with those of da Silva Filomeno et al.^36^, who reported increased body mass in infected high fat diet group compared to an infected standard chow group and in an uninfected high fat diet group compared to a normal group.

Results of the current study showed that the total numbers of worms recovered from the infected obese mice were significantly higher than those recovered from the infected control group, in accordance with Alencar et al.^37^, who reported increased worm burden in the infected high fat diet group, indicating an increase in worm burden associated with modulation of the host lipid metabolism. This could be explained by signalling pathways by which parasites detect host signals and respond to them in a way that both increases the survival and reproduction of the adult worm^14^.

In our animal model of hepatic schistosomiasis, the results revealed higher total tissue egg output in the obesity group than in the control group. These observations were consistent with the findings of Alencar et al.^38^ and da Silva Filomeno et al.^36^, who both reported higher ova count in high-fat chow groups. In contrast, our findings differ from those of Neves et al.^39^, who revealed no statistically significant difference in total intestine egg counts between mice fed a high fat diet and a standard chow diet. These variations could be attributed to experimental approaches such as mouse lineage, dietary diversity, and/or infection time. The increased egg counts in the infected obese group in this study could be explained by the increase in adult count and/or the fecundity of females.

In our study, there was no significant difference in ova counts between the infected control group and infected T1DM group. This agrees with Thabet et al.^40^ and Hulstijn et al.^4^, who observed non-significant difference in tissue ova counts between nondiabetic and STZ-induced diabetic groups using Swiss Webster mice with approximately similar doses of STZ to induce diabetes. These results, however, are not consistent with other studies reporting a reduced tissue egg count in alloxan-induced diabetes in mice^41^.

While egg numbers are an important factor reflecting the potential for disease transmission, the development and maturation of eggs is another consideration of a disease model. The oogram in this study showed no differences in the percentages of dead eggs between groups. Infected groups showed a higher percentage of immature eggs and a lower percentage of mature eggs in the T1DM group. These results support the study by Hulstijn et al.^4^. In our study, the obesity group revealed a higher percentage of mature eggs and a lower percentage of immature eggs, which coincides with Neves et al.^14^. They suggested that a high-fat diet could act as an environmental modulator by facilitating exchanges between miracidium inside the eggs and nutrients discharged mainly in the small intestine^42^.

Interleukin-5 is a key cytokine in circulating and tissue eosinophil differentiation, survival, and persistence, blocking apoptosis and inducing cell activation^43^. IL-5-knockout mice infected with *S. mansoni* produce granulomata that are completely devoid of eosinophils, are smaller, and have less fibrosis than wild-type animals^44^. We presented here IL-5 as elevated in all comorbidity groups when compared to the control group. The soluble egg antigen of *S. mansoni* prevents diabetes in NOD mice by inducing Th2 and Treg responses^45^. Metabolic disorders cause inflammatory responses through Th1 cells, Th17 cells, and classically activated macrophages (CAMs). After infection with *Schistosoma* in T2DM mice, the immunological balance is shifted to Th2 cells, Treg cells, and alternatively activated macrophages (AAMs), as reported by Tang et al.^9^.

In contrast, IL-10 plays a regulatory role in schistosomiasis, preventing the development of excessive pathologies caused by both Th1 and Th2 responses^46^. Obesity increases the B-cell population that is involved in inflammation and insulin resistance in T2DM and obesity^47^. Suppressive B cells and Tregs have the ability to secrete anti-inflammatory cytokines, such as IL-10, to dampen inflammation^48^. Our result of IL-10 levels showed increased levels in infected groups compared to their respective noninfected groups, which is in accordance with the observations of He et al.^49^, Zaccone et al.^50^, and Toulah et al.^51^. Several studies have shown that administration of exogenous IL-10 can prevent the development of insulitis or diabetes in NOD mice^52,53^. Increasing levels of IL-5 and IL-10 over time for hepatic schistosomiasis is a hallmark of the disease^54^.

Tumour necrosis factor beta is predominantly produced by mitogen-stimulated T-lymphocytes and leukocytes. Fibroblasts, astrocytes, myeloma cells, endothelial cells, epithelial cells, and a variety of transformed cell lines all secrete it. Interferons and IL-2 stimulate the synthesis of TNF-β^55^. This cytokine is the main transcriptional regulator of interferon (INF-γ) and is responsible for insulin resistance in response to a high-fat diet^56^. In this study, levels of TNF were significantly increased in the infected control group in relation to the normal group. Moreover, there were significant increases in the infected T1DM, T2DM and obesity groups compared with the infected control group. These increasing levels were antagonized by increasing levels of IL-5, which is a Th2 collagen-induced cytokine, to protect the host. *Schistosoma* infection causes skewing of the immune response towards Th2 and Treg in T2DM-infected mice as described by Tang et al.^9^.

Another important aspect of the host’s response to schistosomiasis is the host cellular response^57,58^. As FOXP3 + T cells can suppress many types of immune cells, such as CD4 + T cells and NK cells, they have the potential to suppress a wide spectrum of immunological diseases, such as diabetes. Infection is a type of disease that is different from autoimmune diseases and the suppressive function of FOXP3 T cells may be disadvantageous for hosts during infection. In infection, some pathogens can suppress immune responses by expanding FOXP3 + T cells, impeding immunopathological injury and limiting protective immunity^59,60^. Indeed, schistosome eggs produce specific molecules that polarize naive CD4 + T cells towards Th2, which has a regulatory function^61^. Our findings regarding FOXP3 coincide with previous findings that reported the induction of CD4 + CD25 + FOXP3 + regulatory T cells due to elevated IL-10, although this finding was associated with impaired immunosuppressive function^49^. Increased activation of FOXP3 has been shown to prevent diabetes in NOD mice^62,63^.

In this study, *Schistosoma* infection caused a skewing of the immune response towards Th2 cytokines; IL-5 and Treg cytokines; IL-10 and FOXP3. The egg induced eosinophils, basophils, and mast cells cause elevation in immunoglobulin E (IgE) levels, suppressing the inflammatory response, and contributing to the formation of granulomas and liver fibrosis^64^.

Pentraxins are an acute-phase protein superfamily that induces either short pentraxins such as C-reactive protein (CRP) or long pentraxins such as PTX3. Chronic hyperglycaemia seems to increase serum PTX3 levels^65^. In the infected groups, there were significant increases in the diabetic and obesity groups compared with the control group and in the infected control group in relation to the normal group.

Infections, non-alcoholic fatty liver disease (NAFLD), and tumours are all associated with elevated pentraxin-3 (PTX3) levels. Serum PTX3 levels appear to reflect an inflammatory response due to changes in the metabolic profile in patients with concomitant NAFLD and hepatitis C virus (HCV) infection^66^. Its level rises rapidly in inflammatory conditions, reaching a peak after 6–8 h of any inflammatory condition; its early rise is due to the rapid release of stored PTX3 by activated neutrophils^67,68^.

Schistosomes are unable to synthesize insulin and human insulin can be bound by insulin receptors. Therefore, a lack of insulin will affect the growth and reproduction of parasites^6^. Insulin levels in the infected groups were significantly decreased in the T1DM group and significantly increased in the obesity group. Our observations regarding insulin levels are in accordance with previous findings^41^, who demonstrated a significant reduction in fasting serum insulin levels in infected mice (50%) compared to negative control mice, while infected DM mice exhibited a 13.6% insulin depression when compared to infected mice.

Adult schistosomes were found to express insulin receptors (IRs), which are necessary for many biological processes including glucose uptake, parasite growth, metabolism, and reproduction. Therefore, a lack of insulin may affect parasite growth and reproduction^6^. Consumption of insulin by the adult worm could be one of the mechanisms for its reduction in infected control groups. Liver damage caused by diabetes and the induction of infection suppress thyroid hormones, which regulate the insulin effect on adipose tissue^41^.

In our study, liver dysfunction caused by fibrosis was more severe in the infected obesity group, explaining a significant decrease in insulin levels compared to the noninfected obesity group. The role of host insulin in the sexual differentiation of juvenile schistosomes has also been suggested^69^. The direct impact on the adult parasite is worthy of future investigation to ascertain the complete interaction between the host, the parasite, and pathology in these disease models.

The adult worms are facultative anaerobes and get most of their energy from carbohydrate degradation from glycogen and glucose^70^. Our research showed that diabetic and obese mice had significantly higher blood glucose levels than control mice, in accordance with previous findings^36^. The increased glucose levels in these groups could be derived from glycogenolysis and/or gluconeogenesis^71,72^. Additionally, there was a significant decrease in glucose levels in the infected obesity group compared to the noninfected obesity group, in accordance with previous findings^73^.

Chronic schistosomiasis may have the ability to lower blood sugar levels. This could be related to the reduction of pro-inflammatory cytokine production^74^, stimulation of white adipose tissue eosinophils, and shift of immunological balance into alternate activated macrophage (M2) pathways, which increase insulin sensitivity and preserve glucose homeostasis^73^. Infection with *S. mansoni* can prevent cholesterol esterification in infected livers. Adult worms cannot produce their own cholesterol and must rely on LDL-C from their hosts^51^. Low density lipoprotein binding and internalisation on the schistosomula surface are also mechanisms of alteration^75^. These mechanisms play an important role in this reduction. In our study, the lipid profile was significantly increased in the infected T1DM, T2DM and obesity groups compared with the infected control group. This corresponds to Neves et al.^14^, who reported significantly increased HDL and LDL in an infected high-fat chow group compared to an infected standard chow group. Further, Jiang et al.^76^. reported increased total plasma cholesterol and TG in diabetic mice compared with nondiabetic control mice.

The parasite eggs that become trapped in host tissues, mainly the liver and intestines, are the main cause of schistosomal pathology. Continuous antigenic stimulation from these eggs attracts inflammatory cells to infection sites, resulting in the formation of granulomas^77^. These complex structures have variable size and cellular composition that are largely determined by local variables such as time of infection and host nutritional status. The impact of the type of diet and general parameters like immune cells and cytokine patterns is crucial^78,79^. Exudative, exudative/ exudative productive, and exudative productive granulomas were found in the infected control and T1DM groups in the present study, with formation of granulomas surrounded by a concentric ring of epithelioid cells, eosinophils, plasma cells, macrophages, and lymphocytes. Granulomas from infected T2DM and obese mice displayed productive stages. This difference in cellular constituents in mice fed a high fat diet is due to a greater wealth of cells than in mice fed a standard diet^39^.

Fibrosis is the primary cause of *Schistosoma*-induced pathology. HSCs play an important role in this process. Furthermore, several studies have found a link between the intensity of liver fibrosis and the number of activated stellate cells^80,81^. HSCs can be activated to become collagen-producing myofibroblasts, which are responsible for developing hepatic fibrosis and increasing vascular resistance leading to portal hypertension and disruption of normal liver architecture^82,83^. Grade III GFAP expression was predominant in the control, T2DM and obesity groups. The T1DM group mainly expressed grade II, indicating a lower number of activated HSCs. These findings coincide with a recent study by Abou Rayia et al.^84^, who reported an infected control group with 65% of GFAP-stained HSCs grade III and 40% having grade II-stained cells in a praziquantel-treated group.

There were significant increases in hepatic and intestinal granuloma density/1 mm^2^ of infected obese group compared to the infected control group. There were no significant differences in hepatic granuloma diameter (µm) in the infected diabetic and obese groups compared to the infected control group. There was significant decrease in intestinal granuloma diameter (µm) in infected DM1 group compared to infected control group which is in accordance with Mahmoud^85^ and El-Shenawy and Soliman^41^. This may be explained by the fact that induction of diabetes causes immunosuppression and impairment of cell-mediated immunity due to deficiency in lymphokine production by lymphocyte and insulin receptor dysfunction on the lymphocytes and macrophages^86^.

With image analysis, the fibrosis area percentage increased significantly in the T2DM and obesity groups. On the other hand, the T1DM group showed a lower fibrosis area and integrated density, which may be attributable to decreased egg maturation, productive granulomas, and fibroblast production. Neves et al.^87^ suggested that there is an association between high-fat intake and schistosomiasis that leads to the acceleration and progression of liver injury. Cholesterol may play an active role in controlling the processes of cell signalling, reproduction, and egg laying in worms from mice fed a high-fat diet. As a result, despite shielding the host from obesity and its effects, liver fibrosis may worsen^14^.

Using helminth parasites or their subunits in disease treatment has been suggested by the hygiene hypothesis^88^, in which immunomodulatory products from worms have been considered for clinical purposes. The parasite infection or its products have important roles in ameliorating metabolic and inflammatory disorders; however, their medical application is limited by the complications of *Schistosoma* infection and the potential immunogenicity of their products.

Induction of T2DM and obesity in the present study increased hepatic pathology and fibrosis, as evidenced by increased fibrosis density and increased expression of HSCs. Therefore, an effective anti-*Schistosoma* vaccine is important in eliminating schistosomiasis^89^, especially in areas with high endemicity where multimorbidity of non-communicable diseases and schistosomiasis are the rule rather than the exception.

More research should be done using soluble egg antigen as helminth therapy in T1DM. As we found striking changes of the manifestations of schistosomiasis in obese mice, determinant factors of obesity such as proinflammatory cytokines, leptin, and adiponectin have to be investigated.

## Conclusion

Our study contributes to the understanding of the mechanisms of the interaction between schistosome infection and metabolic disorders in infected hosts. The parasitological, immunological, biochemical, and histopathological parameters of the host were examined in infected T1DM, T2DM, and obese mice. Induction of T1DM in *S. mansoni*-infected mice caused a decrease in body mass and mature egg percentage. Also, increased levels of TNF- β, IL-5, and PTX3 were demonstrated. It exerted a marginal effect on the liver pathology, as evidenced by the absence of statistical differences in the granuloma diameter, granuloma density and fibrosis area percentage compared to the infected control mice with a predominance of exudative/exudative-productive (E/EP) type of granuloma. Also, liver fibrosis was reduced, as evidenced by reduction in collagen deposition and counts of HSCs. Moreover, there was evidence of a switch of the immune response towards Th2 and Treg pathways (increased levels of IL-5, IL-10, and FOXP3).

Induction of T2DM caused an increase in body mass and mature egg percentage. It increased hepatic pathology and fibrosis, as evidenced by increased fibrosis density with a predominance of exudative-productive (EP) type of granuloma, and increased expression of HSCs. Also, increased levels of TNF- β, IL-5 and PTX3 were demonstrated. Induction of obesity increased parasite burden, as evidenced by an increase in adult worm load, tissue egg counts and mature egg percentage. It also increased hepatic pathology and fibrosis, as evidenced by increased granuloma density and fibrosis density with a predominance of productive (P) type of granuloma as well as increased expression of HSCs. In common with our experimental diabetic models, there was a shift of the immune response towards Th2 and Treg pathways (increased levels of IL-5, IL-10, and FOXP3). Also, evidence of increased systemic inflammation was evident in both schistosomiasis and obesity; however, no further increase in systemic inflammation was observed in combined comorbidities.

Schistosome infection caused an increase in Th2 and regulatory T-cell cytokines and induced changes in lipid profile and blood glucose levels in infected diabetic and obesity groups. Also, it impacted favorably insulin levels in obese mice. More research comparing immunological markers in tissues and utilizing IL inhibitors is recommended to reveal the full degree of alterations. By better understanding the complexities of host–parasite interactions, efforts to reduce the burden of these debilitating diseases can be improved.

## Supplementary Information

Supplementary Tables.

## Data Availability

The datasets generated during the current study are available from the corresponding author on reasonable request.

## Abbreviations

*S. mansoni*: *Schistosoma mansoni*
STZ: Streptozotocin
T1DM: Type 1 diabetes mellitus
T2DM: Type 2 diabetes mellitus
TNF: Tumour necrosis factor
Th: T-helper
IL: Interleukin
FOXP3: Forkhead box P3
Tregs: Regulatory T cells
PTX3: Pentraxin 3
GFAP: Anti-glial fibrillary acidic protein
HSCs: Hepatic stellate cells
NOD: Nonobese diabetic
LDL: Low-density lipoprotein
HDL: High-density lipoproteins
TG: Triglycerides
TC: Total cholesterol
OD: Optical density
