# Supplementary material for: The interaction of Schistosoma mansoni infection with diabetes mellitus and obesity in mice
Source: Sci Rep. 2023 Jun 9;13:9417. doi: 10.1038/s41598-023-36112-5 (PMC10256771; doi:10.1038/s41598-023-36112-5)
Supplement: Supplementary file 1 — Supplementary Tables. [file 41598_2023_36112_MOESM1_ESM.pdf]

The interaction of *Schistosoma mansoni* infection with diabetes mellitus and obesity in mice

Alaa S. Amer[1*], Ahmad A. Othman[1], Lamees M. Dawood[2], Kholoud A. El-Nouby[1], Geoffrey N. Gobert[3]and Dina M. Abou Rayia[1]

[1]Medical Parasitology Department, Faculty of Medicine, Tanta University, Tanta, 31527, Egypt

[2]Biochemistry Department, Faculty of Medicine, Tanta University, Tanta, 31527, Egypt

[3]School of Biological Science, Institute for Global Food Security, Queen's University Belfast, Belfast, BT9 5DL, UK

*Correspondence: alaa.saed@med.tanta.edu.eg

Email addresses

AA: alaa.saed@med.tanta.edu.eg AO: ahmed_ali44@hotmail.com LD: lamees.dawood@med.tanta.edu.eg KE: kh_04@hotmail.com GG: g.gobert@qub.ac.uk DA: dina_aboraya@yahoo.com

Supplementary information

Additional file 1: Table S1. Quantification of the collage fibers from the region of interest in the infected groups using Fiji's deconvolution plugin.

Additional file 2: Table S2. Immunohistochemical expression of GFAP of hepatic stellate cells (HSCs) in livers of infected and control groups.

Additional file 1: Table S1. Quantification of the collage fibers from the region of interest in the infected groups using Fiji's deconvolution plugin. There were significant differences when comparing the T2DM and obesity groups with the control group, $^{**}P < 0.01$, $^{****}P < 0.0001$. (n=10 per group). T1DM, type 1 diabetes mellitus; T2DM, type 2 diabetes mellitus.

Groups	Infected control			Infected T1DM			Infected T2DM			Infected obesity		
image analysis	Area	IntDen	Area%	Area	IntDen	Area%	Area	IntDen	Area%**	Area	IntDen	Area%****
Minimum	905	222562	0.337	609	150131	0.525	8610	325942	2.266	5973	1387241	4.875
Maximum	97137	23160728	4.831	14552	3514348	7.231	135423	16136788	14.83	210934	25023210	24.03
Range	96232	22938166	4.494	13943	3364217	6.706	126813	15810846	12.56	204961	23635969	19.15
Mean	26581	6386451	2.3	4864	1184350	2.776	53046	6990882	8.808	52126	7563301	10.85
Std. Deviation	29793	7088376	1.345	4940	1197598	2.002	39022	6387489	4.234	60529	7117655	5.336
Std. Error of Mean	9421	2241541	0.425	1562	378714	0.6393	12340	2019901	1.342	19141	2250800	1.687

Additional file 2: Table S2. Percentages of immunohistochemical expression of GFAP of hepatic stellate cells (HSCs) in livers of infected and control groups. Semiquantitative grading of GFAP immunostained HSCs: 0: no staining or less than 3% staining; I: 3-33 % positive staining; II: 34-66 % positive staining; and III: more than 66 % positive staining. There were no significant differences between control and infected groups, $P > 0.05$. (n=10 per group). T1DM, type 1 diabetes mellitus; T2DM, type 2 diabetes mellitus.

Groups	0	I	II	III
Infected control	0	20	30	50
Infected T1DM	0	30	40	30
Infected T2DM	0	10	40	50
Infected obesity	0	0	40	60
